# Supplementary material for: The Synergy between Glutathione and Phenols—Phenolic Antioxidants Repair Glutathione: Closing the Virtuous Circle—A Theoretical Insight
Source: Antioxidants (Basel). 2023 May 19;12(5):1125. doi: 10.3390/antiox12051125 (PMC10215641; doi:10.3390/antiox12051125)
Supplement: Supplementary file 1 [file antioxidants-12-01125-s001.zip › antioxidants-2380873-supplementary.pdf]

Supplementary Materials for:

The Synergy between Glutathione and Phenols—Phenolic Antioxidants Repair Glutathione: Closing the Virtuous Circle—A Theoretical Insight

Mirzam Carreon-Gonzalez and Juan Raúl Alvarez-Idaboy *

Departamento de Física y Química Teórica, Facultad de Química, Universidad Nacional Autónoma de México, Mexico City 04510, Mexico

Contents:

SOMO and HOMO orbitals at the TS geometries of the GS$^{\bullet}$ radical and piceatannol reaction: Figure S1.

Optimized molecular geometries of the transition states of GS$^{\bullet}$ radical and piceatannol reaction in Cartesian coordinates (Angstroms): Table S1

SOMO and HOMO orbitals at the TS geometries of the GS• radical and piceatannol reaction:

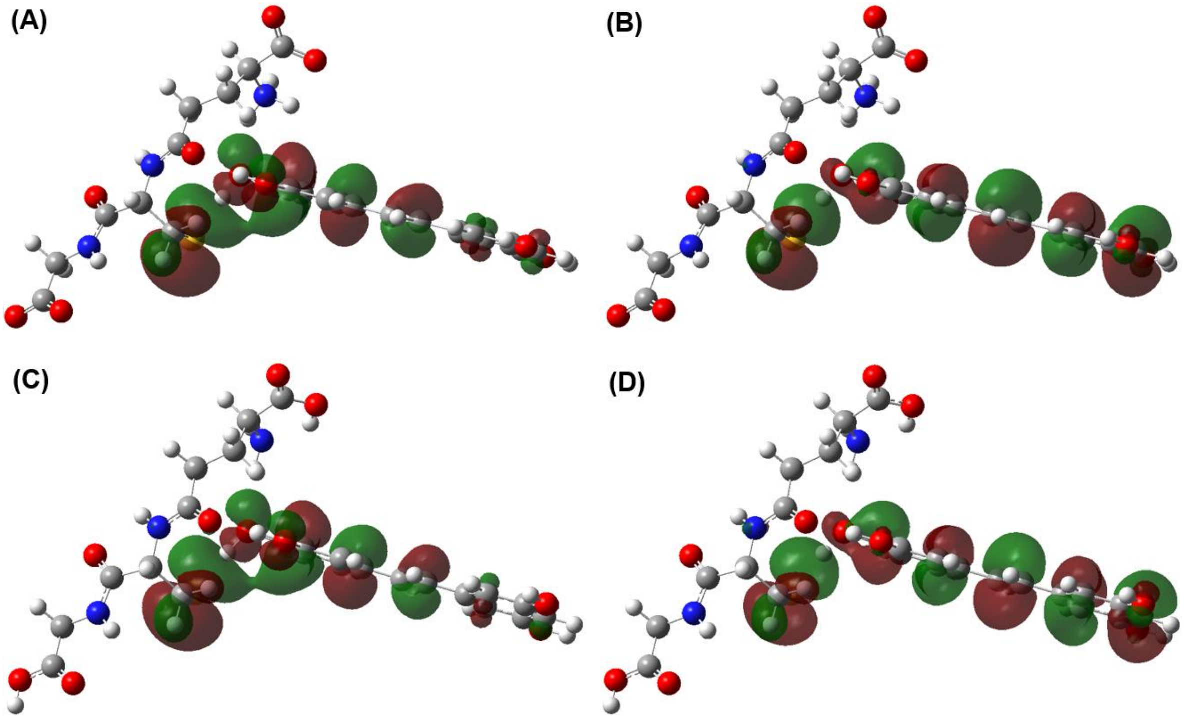

Figure S1. Molecular orbitals. (A) SOMO and (B) HOMO density surfaces at the transition state geometry for the proton coupled–electron transfer (PCET) reaction between the GS• radical and piceatannol in aqueous medium, (C) and (D) are respectively the SOMO and HOMO in lipid medium. Computed with isodensity value of 0.02 a.u

Table S1. Optimized molecular geometries of the transition states of GS• radical and piceatannol reaction in Cartesian coordinates (Angstroms):

Structure for GS•-piceatannol transition state in aqueous medium, optimized at M06-2X/ 6-311++ G(d,p) level:

C	-3.47113800	-1.30427000	0.54382500
H	-2.66176200	-1.00453700	1.21078100
H	-3.82732500	-2.27595800	0.89421900
S	-2.80190900	-1.53208000	-1.12818300
H	-1.73742300	-0.11786400	-1.01100900
O	-1.11145400	0.69190900	-0.69279100
C	0.14836500	0.31653400	-0.47496000
C	0.60545700	0.18972400	0.86214100
C	1.03810400	0.07025400	-1.53772500
C	1.93131200	-0.14539800	1.10082200
C	2.34395500	-0.26908200	-1.29119800
H	2.27097000	-0.22829800	2.12718000
H	3.00927100	-0.45407300	-2.12425900
C	-4.60770500	-0.27132700	0.62372700
H	-4.91424700	-0.19018700	1.67085700
C	-5.81620500	-0.71255300	-0.20143400
O	-6.18475300	-0.07876600	-1.19494800
N	-6.42434200	-1.82077000	0.21858600
H	-6.09939200	-2.31705700	1.04130300
C	-7.55243300	-2.39986900	-0.48224000
H	-8.39307600	-1.70179800	-0.50329900

H	-7.28575500	-2.62480500	-1.51786900
C	-8.01534000	-3.69431400	0.19123100
O	-7.40278200	-4.07894900	1.21512000
O	-8.98925100	-4.26733600	-0.35438300
N	-4.20107900	1.03240600	0.13975700
C	-3.27124300	1.78042700	0.74503800
H	-4.53580800	1.29792700	-0.77943400
O	-2.77806300	1.45779800	1.83605800
C	-2.87363300	3.04673900	0.02027400
H	-2.87216500	2.83279600	-1.05219000
H	-3.66445000	3.78530600	0.18169800
C	-1.53221400	3.60429200	0.50109500
H	-0.86447900	2.79942800	0.82053400
H	-1.68498600	4.25267300	1.36412200
C	-0.81085900	4.41192300	-0.57440200
H	-1.48811100	5.09588800	-1.08421600
C	0.35742800	5.20029700	0.05074700
O	0.04106600	6.23671400	0.67159400
O	1.50369700	4.71257600	-0.08496200
N	-0.24350900	3.48010100	-1.60023400
H	-0.84963400	2.67375700	-1.78757400
H	-0.05999300	3.96385600	-2.48239100
O	-0.21529100	0.38863800	1.90632500
H	-1.10266700	0.69760000	1.62948200
H	0.65854400	3.13617400	-1.24287300

H	0.65905800	0.16104200	-2.54954600
C	2.81675300	-0.37698000	0.04440300
C	4.19400700	-0.72383200	0.36763200
H	4.40701300	-0.80560400	1.42945700
C	5.17420400	-0.93859400	-0.52937600
H	4.96152500	-0.85872700	-1.59130300
C	6.56160700	-1.29103800	-0.21433700
C	7.04537900	-1.36512300	1.10067200
C	7.41836400	-1.56181900	-1.28470300
C	8.37077500	-1.71259700	1.31737200
H	6.41110400	-1.15155800	1.95316300
C	8.74405400	-1.91071000	-1.03955100
H	7.06016200	-1.50663800	-2.30606700
C	9.23417500	-1.98895700	0.25700700
O	8.90056300	-1.80043300	2.57670100
O	9.53638300	-2.16943300	-2.12489900
H	10.26740700	-2.25703800	0.44820300
H	8.22400500	-1.58659400	3.23180000
H	10.42534000	-2.40870500	-1.83348800

Structure for GS•-piceatannol transition state in lipid medium (pentylethanoate), optimized at M06-2X/6-311++ G(d,p) level:

C	-3.29113000	-1.35716600	0.33065400
H	-2.39883200	-1.13702200	0.91801400
H	-3.56700700	-2.39055900	0.55574500

S	-2.87735300	-1.26877700	-1.43317900
H	-1.74633900	0.09386200	-1.25448600
O	-1.05425500	0.83967800	-0.92020400
C	0.20407100	0.40472200	-0.89552100
C	0.85640300	0.26490600	0.36379900
C	0.90416000	0.10486600	-2.07601600
C	2.18977200	-0.12245600	0.40030800
C	2.21596100	-0.29582400	-2.01993200
C	2.88825000	-0.40747200	-0.77495600
H	2.65690000	-0.20905000	1.37335900
H	2.75467400	-0.52551800	-2.93195200
C	-4.41696400	-0.39775100	0.75779200
H	-4.53267000	-0.47734800	1.84430100
C	-5.75302300	-0.75597300	0.10106600
O	-6.33395200	0.01098900	-0.64920600
N	-6.23220400	-1.97607300	0.40918800
H	-5.75840300	-2.57456600	1.07396200
C	-7.47267400	-2.44963700	-0.15303000
H	-8.30293700	-1.77721200	0.08275800
H	-7.41491100	-2.52397300	-1.24321000
C	-7.78168100	-3.81332800	0.40601400
O	-7.09194700	-4.40456700	1.19381900
O	-8.92628000	-4.29150500	-0.08346800
H	-9.08381800	-5.16878400	0.29986400
N	-4.12786000	0.97373700	0.40066600

C	-3.08404400	1.65581100	0.89477300
H	-4.68737200	1.36684200	-0.34762600
O	-2.36481100	1.19088000	1.78171400
C	-2.85175100	3.01809000	0.27203000
H	-2.91353800	2.89329300	-0.81349000
H	-3.68277400	3.67097200	0.55725800
C	-1.52075300	3.64377400	0.67908300
H	-0.75110000	2.87322400	0.78221900
H	-1.61056600	4.13510500	1.64829200
C	-1.03112400	4.65463400	-0.36295500
H	-1.82383200	5.37861500	-0.57454500
C	0.13246800	5.45118700	0.24265900
O	-0.00394600	6.22010500	1.15770700
O	1.31081400	5.20348600	-0.31730000
H	1.11043000	4.57039300	-1.04642800
N	-0.57059700	3.97564200	-1.57750400
H	-0.83805700	2.99551600	-1.59109700
H	-0.92310800	4.41258500	-2.42156300
O	0.23052400	0.50287200	1.51427900
H	-0.71107700	0.77050300	1.41649800
C	4.28519300	-0.82385800	-0.79632000
H	4.67380400	-1.05570900	-1.78342200
C	5.09336200	-0.92366500	0.27419500
H	4.70920800	-0.67407400	1.25943100
C	6.49986800	-1.33775900	0.25430300

C	7.20332200	-1.30645800	1.46563500
C	7.14923000	-1.75413600	-0.91162600
C	8.54219200	-1.68253700	1.50080400
H	6.70388800	-0.98559100	2.37470000
C	8.48692100	-2.12693700	-0.85514400
H	6.64217600	-1.80023300	-1.86651100
C	9.19569100	-2.09464300	0.34373000
H	10.24013700	-2.38453500	0.39074600
O	9.27113100	-1.66861900	2.64632200
H	8.72392600	-1.36101300	3.37818600
O	9.06882600	-2.52356500	-2.01661300
H	9.99086300	-2.75750400	-1.85988600
H	0.38147900	0.20068900	-3.02116700

Geometries of H-bonded transition states in water and pentylethanoate for GS• radical and piceatannol reaction:

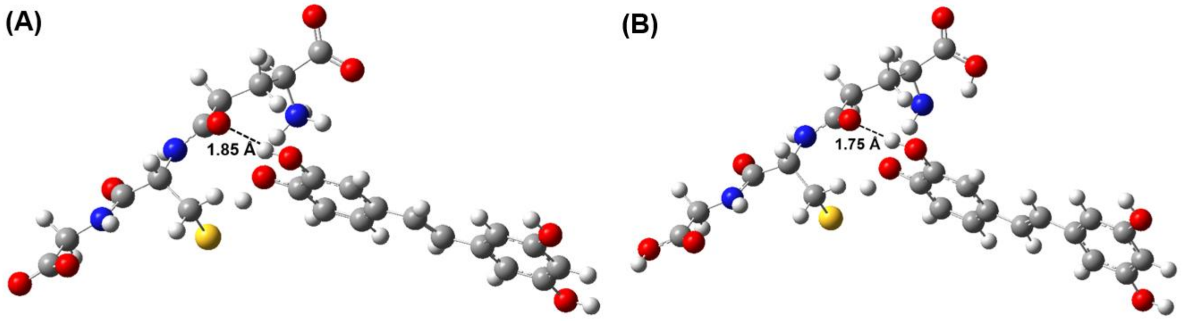

Transition states with H-bond for the reaction of GS• radical and piceatannol in (A) an aqueous and (B) a lipid medium.

Graphics of the evolution of the NPA atomic charges and Hirshfeld spin densities as functions of the IRC for the GS$^\bullet$ radical and piceatannol reaction:

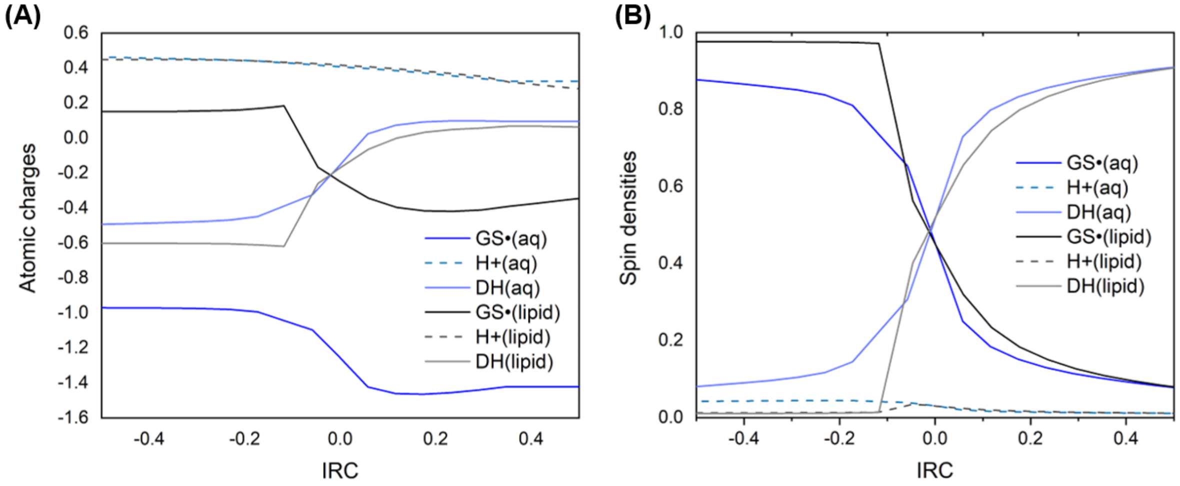

Evolution of NPA atomic charges and Hirshfeld spin densities for the H-acceptor (GS$^\bullet$), H-transferred (H^+) and H-donor (DH=piceatannol) as functions of the intrinsic reaction coordinate (IRC) for the proton–coupled electron transfer (PCET) reaction between the GS$^\bullet$ radical and piceatannol in aqueous (blue) and lipid (black) medium.
